# Turkish *Scorzonera* Species Extracts Attenuate Cytokine Secretion via Inhibition of NF-κB Activation, Showing Anti-Inflammatory Effect *in Vitro*

**DOI:** 10.3390/molecules21010043

**Published:** 2015-12-30

**Authors:** Özlem Bahadır Acikara, Jan Hošek, Petr Babula, Josef Cvačka, Miloš Budešínský, Martin Dračinský, Gülçin Saltan İşcan, Daniela Kadlecová, Ludmila Ballová, Karel Šmejkal

**Affiliations:** 1 Department of Pharmacognosy, Faculty of Pharmacy, Ankara University, 06100 Ankara, Turkey; bahadir-ozlem@hotmail.com (Ö.B.A.); gulcin.saltan@pharmacy.ankara.edu.tr (G.S.İ.); 2 Department of Molecular Biology and Pharmaceutical Biotechnology, Faculty of Pharmacy, University of Veterinary and Pharmaceutical Sciences Brno, Palackého tř. 1946/1, 61242 Brno, Czech Republic; hosekj@vfu.cz; 3 Department of Natural Drugs, Faculty of Pharmacy, University of Veterinary and Pharmaceutical Sciences Brno, Palackého tř. 1946/1, 61242 Brno, Czech Republic; babulap@vfu.cz (P.B.); danniives@gmail.com (D.K.); 4 Mass Spectrometry Group, Institute of Organic Chemistry and Biochemistry, v.v.i., Academy of Sciences of the Czech Republic, Flemingovo nám. 2, 166 10 Prague 6, Czech Republic; cvacka@uochb.cas.cz; 5 NMR Laboratory, Institute of Organic Chemistry and Biochemistry, v.v.i., Academy of Sciences of the Czech Republic, Flemingovo nám. 2, 166 10 Prague 6, Czech Republic; budesinsky@uochb.cas.cz (M.B.); dracinsky@uochb.cas.cz (M.D.); 6 Department of Pharmacognosy and Botany, The University of Veterinary Medicine and Pharmacy in Košice, Komenského 73, 041 81 Košice, Slovakia; ludmila.ballova@gmail.com

**Keywords:** anti-inflammatory activity, IL-1β, NF-κB, *Scorzonera*, phenolic, TNF-α, triterpen

## Abstract

*Scorzonera* species are used in different folk medicines to combat many diseases, including the illnesses connected with inflammation. Previous experiments showed anti-inflammatory activity of *Scorzonera* extracts *in vivo*. *S. latifolia*, *S. cana* var. *jacquiniana*, *S. tomentosa*, *S. mollis* ssp. *szowitsii*, *S. eriophora*, *S. incisa*, *S. cinerea*, and *S. parviflora* extracts were, therefore, evaluated for their inhibitory activities of TNF-α and IL-1β production, and NF-κB nuclear translocation in THP-1 macrophages. The HPLC analysis was carried out to elucidate and to compare the composition of these extracts. Major compounds of the tested extracts have been isolated using different chromatographic techniques and further tested for their inhibitory activities on TNF-α and IL-1β production. Several extracts showed promising anti-inflammatory activity in these *in vitro* tests. Results of HPLC analysis revealed chlorogenic acid as a compound present in all tested extracts. Hyperoside, quercetin-3-*O*-β-d-glucoside and rutin were also present in varying amount in some *Scorzonera* species analyzed. Furthermore, eight phenolics which were identified as quercetin-3-*O*-β-d-glucoside (**1**), hyperoside (**2**), hydrangenol-8-*O*-glucoside (**3**), swertisin (**4**), 7-methylisoorientin (**5**), 4,5-*O*-dicaffeoyl-quinic acid (**6**), 3,5-di-*O*-caffeoyl-quinic acid (**7**), and chlorogenic acid (**8**) have been isolated as major phenolic compounds of the tested extracts and, together with eight terpenoids (**9**–**16**) previously obtained from different *Scorzonera* species, have been tested for the inhibition of TNF-α production, unfortunately with no activity comparable with standard.

## 1. Introduction

Inflammation is a coordinated and complex biological reaction involving various pro-inflammatory and anti-inflammatory cellular proteins, enzymes, and cytokines [1,2,3,4,5]. Among the pro-inflammatory cytokines, tumor necrosis factor-α (TNF-α) and interleukin 1-β (IL-1β) have a wide range of biological activities on numerous cell types and are reported to be involved in the pathogenesis of various inflammatory disorders, such as rheumatoid arthritis, inflammatory bowel disease, osteoarthritis, psoriasis, endotoxemia, and/or toxic shock syndrome, different types of cancer, and degenerative diseases of the central nervous system [3,6,7]. In response to infection or injury, these cytokines are released by tissue macrophages, and serve to recruit circulating neutrophils to the site of inflammation. They may also directly modulate neutrophil functions across the vascular endothelium [6]. TNF-α, which is reported as a key cytokine in the inflammation, has a wide range of functions for maintaining the normal cellular physiology such as triggering of apoptosis, influence on secretion of cytokines e.g., IL-1, IL-6 and IL-10 as well as activation of T cells and other inflammatory cells [3,8]. TNF-α also causes further activation of the transcription factors NF-κB [9] which regulates about 200 immune, growth, and inflammation-related genes [4]. It is well established that inappropriate and prolonged activation of NF-κB has been linked to several diseases while normal activation of NF-κB is required for cell survival and immunity. Due to the pathophysiological importance of an enhanced production of inflammatory mediators through NF-κB activation, selective inhibitors of NF-κB activation may have broad application as novel therapeutics, for example, anti-inflammatory and anticancer agents [10,11].

Plants used in folk medicine serve as one of the main sources of drug discovery and development [1]. In Turkey, numerous plant species are known for their therapeutic properties and have been used in traditional Turkish folk medicine to treat a wide range of diseases. Plants from the genus *Scorzonera*, belonging to Asteraceae family, are used as food as well as medicinal plants not only in Turkey, but also in some other European countries [12,13,14]. Different species of *Scorzonera* have been used in European traditional medicine against pulmonary diseases, colds, for the treatment of wounds as well as for their stomachic, diuretic, galactagogue, antipyretic, and appetizing effects [12,13,15]; in Mongolian traditional medicine for the treatment of diarrhea, lung edema, parasitic diseases, and fever caused by bacterial, and viral infections [13]; in Libyan folk medicine for the treatment of hepatic pains [16]; and in Chinese, as well as in Tibetan folk medicine against breast inflammation and abscess due to their antipyretic and anti-inflammatory activities [17]. Additionally, in Turkish folk medicine different species of this genus have been reported to be used in treatment of rheumatism, pain, wound healing, as well as arteriosclerosis, kidney diseases, hypertension, and diabetes [18,19].

The extracts from some *Scorzonera* species showed hepatoprotective activity *in vivo* in CCl_4_ induced liver damage in rats and also anti-ulcerative effect (acetic acid-induced gastric ulcer in rats), showing decrease of inflammatory markers during microscopic evaluation of stomach tissue [20]. In order to verify traditional usage of *Scorzonera* species, anti-nocieptive, anti-inflammatory, and wound healing activities have been evaluated and promising results have been obtained in our previous research [21,22,23,24]. The aim of the current study was to evaluate the ability of *Scorzonera* extracts to inhibit TNF-α and IL-1β production and NF-κB nuclear translocation in LPS-stimulated THP-1 macrophages, which may be responsible for the observed anti-inflammatory activity. Eight different *Scorzonera* species: *S. cana* (C.A. Meyer) Hoffm. var. *jacquiniana* (W. Koch) Chamb., *S. cinerea* Boiss., *S. eriophora* DC., *S. incisa* DC., *S. mollis* Bieb. ssp. *szowitzii* (DC.) Chamb., *S. latifolia* (Fisch. and Mey.) DC., *S. parviflora* Jocq., and *S. tomentosa* L., which displayed potent anti-inflammatory activity *in vivo* test models in our previous researches were selected for activity tests [22,23,24]. HPLC analysis of the tested *Scorzonera* extracts was also performed with aim to elucidate their composition. HPLC analytical method previously published by [22] was optimized and composition of the extracts was tested qualitatively to select main compounds for activity testing. According to this and previous work, eight phenolics: quercetin-3-*O*-β-d-glucoside (**1**), hyperoside (**2**), hydrangenol-8-*O*-glucoside (**3**), swertisin (**4**), 7-methylisoorientin (**5**), 4,5-*O*-dicaffeoyl-quinic acid (**6**), 3,5-*O*-dicaffeoyl-quinic acid (**7**), and chlorogenic acid (**8**), which were isolated as the major phenolic compounds in current study, and terpenoids **9**-**16**, which have been isolated previously [taraxasterol acetate (**9**), lupeol (**10**), lupeol acetate (**11**), β-sitosterol (**12**), 3-β-hydroxy-fern-8-en-7-one-acetate (**13**), urs-12-en-11-one-3-acetyl (**14**), 3-β-hydroxy-fern-7-en-6-one-acetate (**15**), and olean-12-en-11-one-3-acetyl (**16**)] were also tested for their inhibitory effects on the TNF-α and IL-1β production.

## 2. Results and Discussion

This study was focused on determination of TNF-α and IL-1β production as well as NF-κB nuclear translocation inhibitory activities of *S. latifolia* (Fisch. & Mey.) DC., *S. cana* (C.A. Meyer) Hoffm. var. *jacquiniana* (W. Koch) Chamb., *S. tomentosa* L., *S. mollis* Bieb. ssp. *szowitzii* (DC.) Chamb., *S. eriophora* DC., *S. incisa* DC., *S. cinerea* Boiss., *S. parviflora* Jocq. aerial part water/methanolic extracts to reveal, if these mechanisms are playing an important role in the anti-inflammatory activity of tested extracts, which was previously observed.

**Figure 1:**
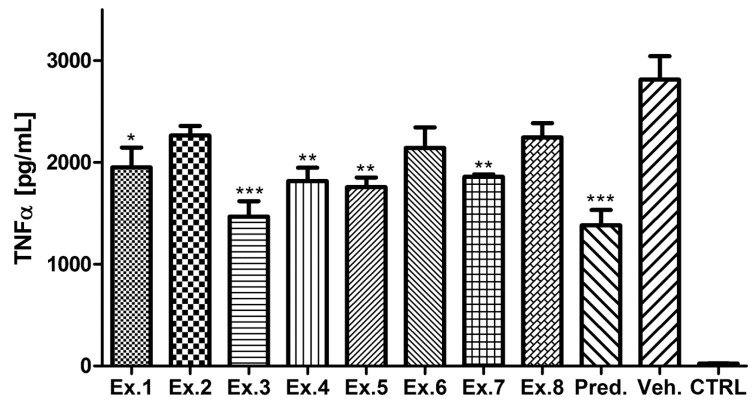
TNF-α production inhibitory activity of *Scorzonera* species. The cells were pretreated with extracts 1–8 (Ex. 1–8) (25 μg/mL), and prednisone (1 µM), or the vehicle (Veh., DMSO) only. After 1 h of the incubation, the inflammatory response was induced by LPS [except of the control cells (CTRL)]. The secretion of TNF-α was measured 24 h after the LPS addition. The results are expressed as mean ± SE for three independent experiments. * Significant difference in comparison to vehicle only treated cells (*p* < 0.05), ** significant difference in comparison to vehicle only treated cells (*p* < 0.01), *** significant difference in comparison to vehicle only treated cells (*p* < 0.001).

LPS-activated macrophages were used for testing of the activity of the extracts. As shown in Figure 1, pretreatment of LPS activated THP-1 cells with *Scorzonera* extracts led to inhibition of TNF-α production. When compared with the vehicle-treated group, activities of all extracts, except of Ex. 2, Ex. 6 and Ex. 8 were found to be significant. Among the tested extracts, *S. tomentosa* aerial part extract has been established as the most active. Additionally, *S. latifolia* aerial part extract displayed notable activity.

**Figure 2:**
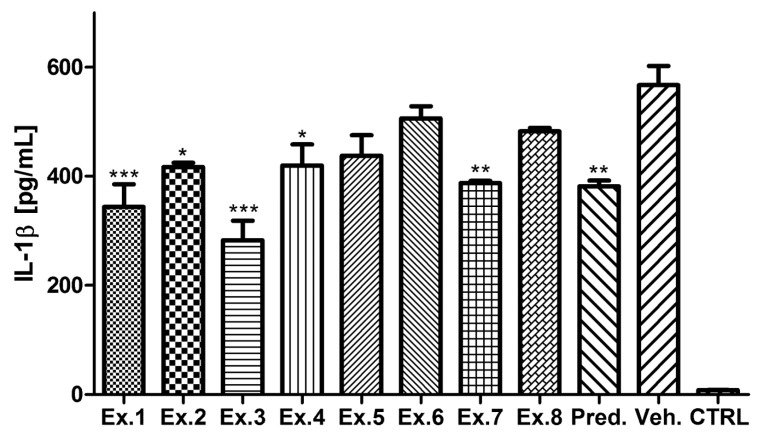
IL-1β production inhibitory activities of *Scorzonera* species. The cells were pretreated with extracts 1–8 (Ex. 1–8) (25 μg/mL), and prednisone (1 µM), or the vehicle (DMSO) only. After 1 h of the incubation, the inflammatory response was induced by LPS [except of the control cells (CTRL)]. The secretion of IL-1β was measured 24 h after the LPS addition. The results are expressed as mean ± SE for three independent experiments. * Significant difference in comparison to vehicle only treated cells (*p* < 0.05), ** significant difference in comparison to vehicle only treated cells (*p* < 0.01), *** significant difference in comparison to vehicle only treated cells (*p* < 0.001).

The ability of the *Scorzonera* extracts to decrease IL-1β production after inflammatory stimulation was also evaluated. Inhibitory activity was observed for all *Scorzonera* extracts tested and results similar to TNF-α production inhibitory test were obtained (Figure 2). When compared with the vehicle-treated group, activities of several extracts, except of Ex. 2, Ex. 5, Ex. 6, and Ex. 8 were found to be significant in testing of IL-1β secretion inhibition.

**Figure 3:**
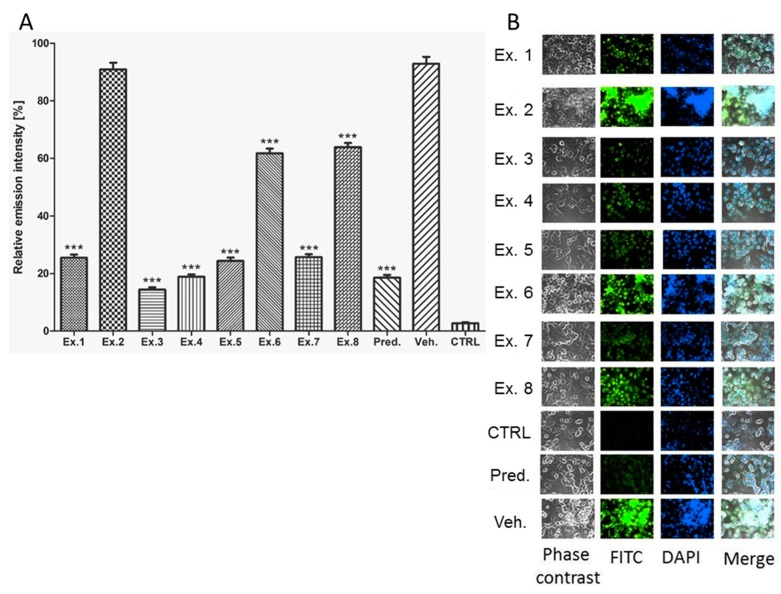
Graphical comparison of inhibitory activity of *Scorzonera* species on NF-κB activation. The cells were pre-treated with extracts 1–8 (Ex. 1–8) (25 μg/mL), and prednisone (1 µM), or the vehicle (DMSO) only. After 1 h of the incubation, the inflammatory response was induced by LPS [except of the control cells (CTRL)]. The NF-κB activation was measured 3 h after the LPS addition. The results are expressed as mean ± SE for three independent experiments (**A**) and quintessential pictures obtained by fluorescent camera (**B**). *** Significant difference in comparison to vehicle only treated cells (*p* < 0.001).

Both cytokines, TNF-α and IL-1β, are under transcription control of NF-κB, which is activated by LPS. As visible from the Figure 3, the effect of *Scorzonera* extracts on NF-κB nuclear activation was evaluated. The anti-p65 antibody used for the assay recognizes the binding site of IκB-α on the p65 protein. The antibody binds to p65 only if the inhibitor IκB-α is degraded and NF-κB could enter to nucleus. In partial accordance with results of TNF-α and IL-1β inhibitory activity testing, Ex. 3, 4, 5, and 7 were assigned to be most potent in inhibition of NF-κB nuclear activation. The results show that *S. tomentosa* aerial part extract has been the most active one.

Later, the analysis of phenolic and terpenoid compounds isolated from *Scorzonera* species was carried out with aim to establish the compound responsible for the effect.

**Figure 4:**
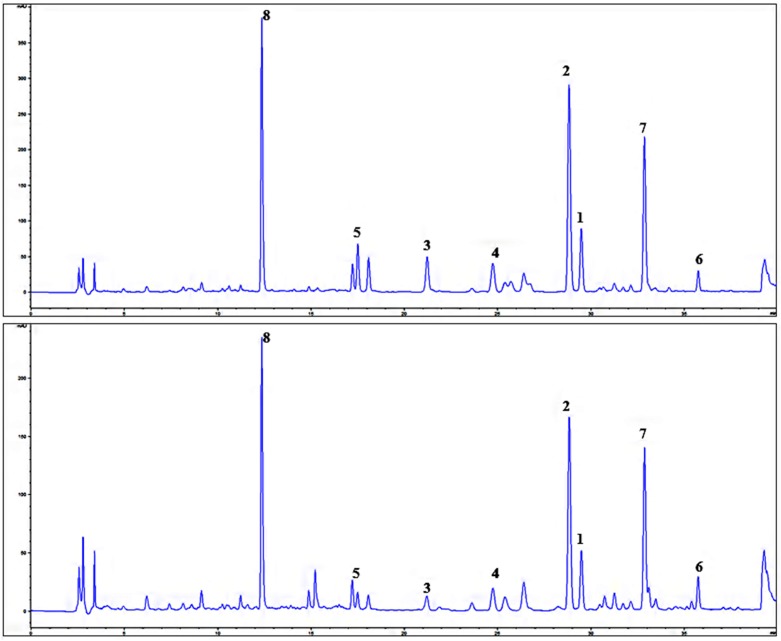
Chromatograms of *S. latifolia* (Ex. 1) and *S. tomentosa* (Ex. 3) aerial part extract; λ 254 nm; quercetin-3-*O*-β-d-glucoside (**1**), hyperoside (**2**), hydrangenol-8-*O*-glucoside (**3**), swertisin (**4**), 7-methylisoorientin (**5**), 3,5-*O*-dicaffeoyl-quinic acid (**6**), 4,5-*O*-dicaffeoyl-quinic acid (**7**), and chlorogenic acid (**8**).

The chromatograms of the extract with greatest NF-κB nuclear translocation inhibitory activity Ex. 1 (*S. latifolia*) and Ex. 3 were (*S. tomentosa*) selected as representative for analysis of Ex. 1–8, and as visible from the Figure 4, compounds **1**–**8** [quercetin-3-*O*-β-d-glucoside (**1**), hyperoside (**2**), hydrangenol-8-*O*-glucoside (**3**), swertisin (**4**), 7-methylisoorientin (**5**), 3,5-*O*-dicaffeoyl-quinic acid (**6**), 4,5-*O*-dicaffeoyl-quinic, acid (**7**) and chlorogenic acid (**8**)] are the main UV-detectable substances observable, which have been later isolated as the major compounds from *S. latifolia* aerial part. According to our knowledge, this is the first phytochemical study related *S. latifolia* aerial part and isolation of swertisin (**4**) as well as 7-methylisoorientin (**5**) from *Scorzonera* species. Further, isolated phenolic compounds **1**–**8** and later also terpenoids **9**–**16** were tested in TNF-α and IL-1β production inhibition assay, compared to effect of prednisone. Unfortunately, no statistically significant activity of these compounds in concentration of 10 µM (lacking cytotoxic effect; Supplementary Materials: Figure S1) has been observed in comparison with prednisone, which was used as the standard.

*Scorzonera* species were in focus of our previous research touching testing of analgesic, anti-inflammatory, and wound healing activities. Aerial part extracts of *Scorzonera* showed significant inhibitory effects in carrageenan and PGE_2_ induced hind paw edema model *in vivo* [21,22,23]. The concentrations of extracts showing the activity was 100 mg/kg of body weight of mice, and the effect was comparable with indomethacine used as the standard at the some concentration. Carrageenan-induced hind paw edema acute inflammation test model is biphasic. First phase starts with the release of histamine, serotonin, and kinins, while the second phase is related to the release of prostaglandins like substances [25]. All of the tested *Scorzonera* extracts were found be inactive in serotonin-induced hind paw edema model [23]. Furthermore, mouse ear edema induced with TPA was also employed for the anti-inflammatory evaluation of *Scorzonera* species and the results have revealed that some *Scorzonera* displayed potent activity while some are completely inactive [23]. Mouse ear edema induced with topically-applied TPA is also an excellent acute inflammation animal model, closely related with the infiltration of neutrophil and macrophages, the induction of pro-inflammatory cytokines including TNF-α and IL-1β, and the generation of ROS including superoxide anion [26]. The anti-inflammatory activity was assayed also by acetic acid-induced capillary permeability test [24] and *S. latifolia*, *S. tomentosa*, *S. mollis* ssp. *szowitsii* showed potent effect. In the present study, extracts of *Scorzonera* species which were found previously to be active in acetic acid-induced capillary permeability test (Ex. 1 and 3) showed notable inhibitory activities on TNF-α and IL-1β production in LPS stimulated THP-1 cell lines. As visible, for example, from a comparison of the concentration used in this experiment with concentrations in previously published studies [27,28], the effect is promising enough to perform further experiments. Moreover, no sign of toxicity of extracts in our *in vitro* assay, and also in previous *in vivo* tests supports the idea of further testing.

Aerial part extracts of the *Scorzonera* species were also investigated for their effects on NF-κB nuclear translocation. The transcription factor NF-κB plays a critical role in inducible expression of genes involved in diverse biological processes, including development, immune and inflammatory responses, cell growth, cell death (apoptosis), and stress responses. NF-κB is found as an inactive form in the cytoplasm in complex bound to its inhibitory subunit IκB. Numerous stimuli activate NF-κB, mostly through IκB kinase-dependent (IKK-dependent) phosphorylation and subsequent degradation of IκB proteins. The released NF-κB dimers translocate to the nucleus, bind to DNA and activate gene transcription. NF-κB regulates a wide variety of important target genes. Among the numerous target genes of NF-κB are those encoding inflammatory and chemotactic cytokines such as interleukin-1 (IL-1), IL-2, IL-6, IL-8, and TNF, cell adhesion molecules, major histocompatibility complex class molecules, cytokine receptors, and pro-inflammatory enzymes such as inducible nitric oxide synthase and cyclooxygenase-2. The activation of NF-κB has been implicated in cancers and in many human chronic inflammatory diseases, such as asthma, arthritis, and inflammatory bowel disease. Therefore, the NF-κB signaling pathway is clearly established as one of most important targets for discovering drugs for the treatment of a wide variety of inflammatory diseases, autoimmune diseases as well as cancer [29,30]. Current study results show that extracts of the *Scorzonera* species (Ex. 1 and 3) inhibited significantly NF-κB activation and consequently TNF-α and IL-1β production at concentration of 25 μg/mL. This concentration was selected according to concentration used in previous assays used for analysis of *Scorzonera* anti-inflammatory potential *in vivo* [23], and it is overall comparable with concentrations of different extract selected as active in literature [31]. Therefore, this assay brings further support of the possible uses of *Scorzonera* in traditional medicines as an anti-inflammatory agent.

HPLC analysis was performed in order to identify the main content compounds of tested extracts (Ex. 1–8). The HPLC method described by Küpeli Akkol *et al.* was improved and used [22]. The results of our analyses confirmed the presence of compounds observed previously (hyperoside (**2**) and chlorogenic acid (**8**) [22,23,24]), and further chromatographic separation and later HPLC analysis also confirmed the presence of other phenols (**1**, **3**–**7**). According to the results, all *Scorzonera* extracts analyzed contain chlorogenic acid (**8**) as one of the main compound [22]. Furthermore, all extracts are found to be rich in flavonoid content such as hyperoside (**2**), rutin, as well as quercetin-3-*O*-β-d-glucoside (**1**). As visible, with exception of **8**, all phenolics identified in *Scorzonera* aerial part extracts are glycosides, in majority flavonoids. The presence of all these compounds (**1**–**8**) in the most active extracts (Ex. 1 and Ex. 3) and previous assays showing relatively high content of phenolics [22,23,24], together with our qualitative HPLC analysis and literature reports about the activity of content compounds lead us to idea of testing the isolated content compounds. Antioxidant, anti-inflammatory, and anti-nociceptive activities of chlorogenic acid (**8**) have previously been reported [2,32,33]. It was isolated as anti-inflammatory compound from *Sambucus ebulus* L. [2] and as anti-inflammatory and analgesic compound from *Cheilanthes farinose* (Forsk.) Kaulf. [33]. An analgesic and anti-inflammatory activity of this compound was also confirmed by Dos Santos *et al.* [32] and chlorogenic acid (**8**) was found to inhibit TNF-α expression in active in reducing the arachidonic acid metabolites, nitric oxide, and pro-inflammatory cytokine production in a dose-dependent manner and under some conditions effect observed was almost comparable to ibuprofen [5]. Thus, it has been suggested that chlorogenic acid (**8**) probably could be one of the compound responsible for the anti-nociceptive and anti-inflammatory activities of *Scorzonera* species. Additionally, flavonoid glycosides and aglycones are mentioned many times as anti-inflammatory principles of food or medicinal plants, with different mode of activity, including the effect on gene expression and production of pro-inflammatory cytokines [33].

Some studies have shown that some flavonoids are modulators of pro-inflammatory cytokine production. Luteolin, apigenin, quercetin, naringenin, genistein were reported to have inhibitory effects on TNF-α production [34,35]. The properties of the flavonoids, such as quercetin and myricetin, may be mediated through down-regulation of the NF-κB pathway [36,37]. The greatest effect on the NF-κB pathway was observed after the treatment of cells with *S. tomentosa* extract (Ex. 3), which contain the highest amounts of chlorogenic acid (**8**) and hyperoside (**2**) among investigated extracts. From the above mentioned, we suggested that chlorogenic acid (**8**), derivatives of dicaffeoyl-quinic acid (**6** and **7**), and flavonoids, especially quercetin-3-*O*-β-d-glucoside (**1**) and hyperoside (**2**) could be responsible for the anti-inflammatory activity of tested extracts. Therefore, further experiments were carried out with phenolic compounds previously obtained from *Scorzonera* species (**1**–**8**), to assay the inhibition of TNF-α and IL-1β production in THP-1 cells, and later also with triterpenes **9**–**16**, because taraxasterol acetate (**9**) isolated from *S. latifolia* showed previously analgesic activity *in vivo* in writhing and tail-flick tests [21] and also other triterpenoid substances like lupeol derivatives (**10** and **11**), β-sitosterol (**12**) and other triterpenes (**13**–**16**) could potentially demonstrate anti-phlogistic effect [38,39]. Unfortunately, only weak activity of compounds tested was observed, and no statistically significant activity has been observed in comparison with standard used (prednisone, Supplementary Materials, Figure S1). However, the activity was assayed at concentration of 10 µM only, literature touching chlorogenic acid (**8**) showed majority of experiments published the usage and effect in substantially greater concentrations (milimolar) [32,33,40], therefore the concentration used by us could be counted as under-dosed, and the effect was not observed. The same can be said for some flavonoids, represented for example by hyperoside (**2**), which previously showed anti-inflammatory effect mediated by decreasing the production of TNF-α and IL-1β [41], but well observable at concentrations greater than 10 μM. Similarly, the activity of flavonoids reported previously [34,35] was observed at concentrations of 50 μM. Touching the triterpenes, our results confirmed the study of Srivastava *et al.* [42] showing low effect of **9** only on LPS-induced neuroinflammation in C6 rat glial cells (low inhibition of TNF-α, IFN-γ, and IL-6 release). However, this compound inhibited the superoxide radical anion generation and elastase release in assay using human neutrophils [43]. This is a good example how the method and concentration used can affect the interpretation of results, even when some anti-inflammatory effects of compounds, which we used for assay, were carried out by different techniques *in vivo*, what makes the comparison with literature more difficult. For us, the important is the comparison of compounds tested (**1**–**16**) with prednisone, which is commonly used as a reference compound, and which showed better effect. Other possibility explaining the discrepancy of results obtained for extracts and compounds tested is the presence of yet undetected substances in extracts, or a synergic activity could be involved. Therefore, further studies on determination of active substances responsible for the TNF-α and IL-1β production inhibition should be carried out.

As conclusion, the anti-inflammatory potential of some *Scorzonera* extracts has been confirmed using *in vitro* TNF-α and IL-1β production inhibition assay on LPS-stimulated THP-1 macrophages, which were supported by inhibition of NF-κB activation. Several compounds isolated from these extracts were further tested for TNF-α and IL-1β production but none of the compounds presented activity. More efforts should be spent on the determination of active substances.

## 3. Experimental Section

### 3.1. Plant Material

*Scorzonera* species were collected in different parts of Anatolia. The taxonomic identification of the plant was confirmed by H. Duman, a plant taxonomist of the Department of Biological Sciences, Faculty of Art and Sciences, Gazi University. Voucher specimens are kept in the herbarium of Ankara University, Faculty of Pharmacy (Table 1).

**Table 1:** Locality of the plant material harvest and extract coding.

Plant Species	Locality	Date	Identification/Voucher Specimen No.	Code Name
*S. latifolia* (Fisch. & Mey.) DC.	Kars, Arpaçay	2005	H. Duman/23830	Ex. 1
*S. cana* (C.A. Meyer) Hoffm. var. *jacquiniana* (W. Koch) Chamb.	Ankara, Çamlıdere	2008	H. Duman/23834	Ex. 2
*S. tomentosa* L.	Yozgat, Akdağmadeni	2005	H. Duman/23841	Ex. 3
*S. mollis* Bieb. ssp. *szowitzii* (DC.) Chamb.	Ankara, Kızılcahamam	2006	M. Koyuncu/23844	Ex. 4
*S. eriophora* DC.	Ankara,Çubuk	2007	H. Duman/23832	Ex. 5
*S. incisa* DC.	Konya, Ermenek	2005	H. Duman/23833	Ex. 6
*S. cinerea* Boiss.	Sivas, Çetinkaya	2005	H. Duman/23829	Ex. 7
*S. parviflora* Jocq.	Ankara, Gölbaşı	2008	H. Duman/25894	Ex. 8

### 3.2. Extraction of Plant Materials for Activity

Dried and powdered aerial parts of the plants (10 g) were separately extracted with 20% aqueous methanol (100 mL) at room temperature for 24 h in three days using continual stirring. Each extract was filtered and concentrated to dryness under reduced pressure and low temperature (40–50 °C) to yield crude extracts (Ex. 1–8).

### 3.3. Isolation and Identification of Compounds

Compounds **1**–**8** were isolated from *S. latifolia* ethyl acetate extract (Supplementary Materials, Figure S2). Aerial part extract of the *S. latifolia* was selected due to its anti-inflammatory activity potential and sufficient quantity. Dried and powdered aerial parts of the plant (1.5 kg) were macerated in methanol (2.5 L × 5) at room temperature for 24 h. The extract was filtered and MeOH removed under reduced pressure and 40–50 °C using a rotary evaporator to get crude extract (231 g). This methanol extract was subjected to liquid-liquid fractionation. *n*-Hexane, chloroform and ethyl acetate were used. Including water part, four fractions of *S. latifolia* extract were obtained. The ethyl acetate part (21.5 g) was used for further separation by column chromatograpy. Elution was performed on silica gel (40–63 μm, Merck) column with EtOAc:MeOH:Water (100:13.5:10, *v*/*v*/*v*) solvent system to obtain 92 subfraction (120 mL). **1** was purified from subfraction 18–19 by preparative TLC on silica gel plates (Merck 5744) using EtOAc:MeOH:Water (70:13.5:10, *v*/*v*/*v*) as mobile phase. Yellow precipitate was occurred in subfraction 21, later identified as **2**. Compounds **3** and **4** were obtained from subfraction 23–24 and 30–31 in crystalline form. Subfraction 41 gave **5** as white amorphous precipitate. Substances **6**–**8** were obtained on reverse phase TLC plates (Merck 5559) with MeOH: Water (1:1, *v*/*v*) solvent system as eluent from subfraction 60–64 and 86–92 respectively. Their identification was carried out using HRMS and ^1^H- and ^13^C-NMR and comparison of data obtained with that in literature [44,45,46]. The exact masses were measured using LTQ Orbitrap XL hybrid mass spectrometer (Thermo Fisher Scientific, Waltham, MA, USA) equipped with an electrospray ion source. The mobile phase consisted of methanol/water (4:1), flow rate of 100 µL/min, and the samples diluted with the mobile phase were injected using a 2-µL loop. The mass spectra were internally calibrated using protonated phthalic anhydride or deprotonated stearic acid as a lock mass. The ^1^H- and ^13^C-NMR spectra were measured on a Bruker AVANCE-600 spectrometer (^1^H at 600.13 MHz, ^13^C at 150.9 MHz) using cryo-probe (5 mm CPTCI ^1^H-^13^C/^15^N/D Z-GRD) in DMSO-*d*_6_ at 298 K. Structural assignment of proton and carbon signals was achieved using 2D-H,H-COSY, 2D-H,H-ROESY, 2D-H,C-HSQC, and 2D-H,C-HMBC spectra.

### 3.4. HPLC Analysis

**Figure 5:**
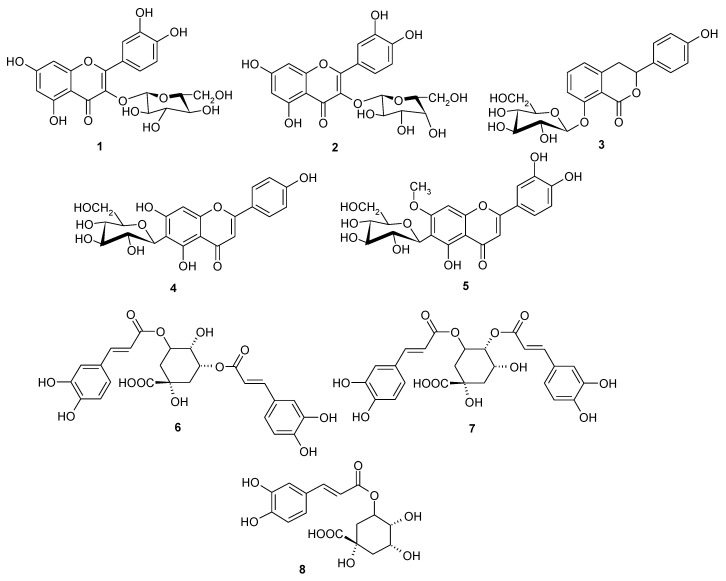
Phenolic constituents of *Scorzonera* species found in *Scorzonera* extracts and tested for TNF-α production inhibition.

HPLC analyses were carried out using Agilent LC 1100 chromatograph (Agilent Technologies, Darmstadt, Germany). The diode array detector (DAD) was set at wavelength of 254 nm and peak areas were integrated automatically using Agilent ChemStation Software. Separation was carried out using a Supelco Ascentis^®^ (Bellefonte, Pennsylvania, PA, USA) Express RP-Amide (150 mm × 4.6 mm; 2.7 μm) column. The mobile phase was composed of acetonitrile (A) and 0.2% HCOOH (B) using gradient elution: initial A:B (8:92, *v*/*v*), in 10th min A:B (18:82), in 20th min. A:B (20:80, *v*/*v*), in 30th min 30:70 (*v*/*v*). This was followed isocratic flow of A:B (30:70, *v*/*v*) to 45th min. The flow rate was 0.5 mL/min, column temperature was maintained at 41 °C. The sample injection volume was 10 μL. The identification of compounds in extracts was carried out using the comparison of retention time and UV spectrum obtained from the analysis of single compounds previously isolated or obtained from commercial sources (**1**–**8**, MeOH solutions, Figure 5). Furthermore, a combined injection of single compound with extract was used to confirm the compound presence.

### 3.5. Anti-Inflammatory Assay

#### 3.5.1. Compounds for *In Vitro* Anti-Inflammatory Assays

**Figure 6:**
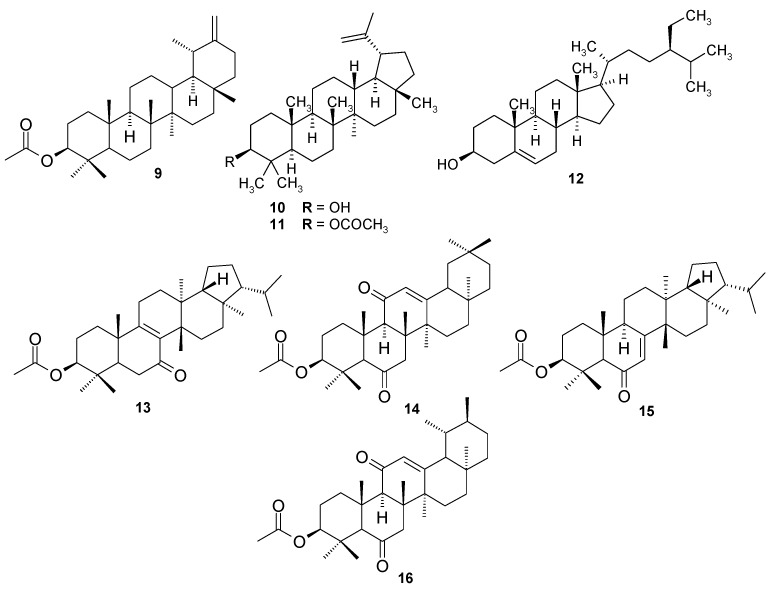
Terpenoid constituents of *Scorzonera* species found in *Scorzonera* extracts and tested for TNF-α production inhibition.

Phenolic compounds **1**–**8** [quercetin-3-*O*-β-d-glucoside (**1**), hyperoside (**2**), hydrangenol-8-*O*-glucoside (**3**), swertisin (**4**), 7-methylisoorientin (**5**), 3,5-*O*-dicaffeoyl-quinic acid (**6**), 4,5-*O*-dicaffeoyl-quinic acid (**7**), chlorogenic acid (**8**)] (Figure 5) and terpenoids **9**–**16** [taraxasterol acetate (**9**), lupeol (**10**), lupeol acetate (**11**), β-sitosterol (**12**), 3-β-hydroxy-fern-8-en-7-one-acetate (**13**), urs-12-en-11-one-3-acetyl (**14**), 3-β-hydroxy-fern-7-en-6-one-acetate (**15**), olean-12-en-11-one-3-acetyl (**16**)] (Figure 6) used for assays were isolated from *Scorzonera* species according to the procedures showed in [47,48,49].

#### 3.5.2. TNF-α and IL-1β Inhibitory Activity Measurement

The RPMI 1640 medium and the penicillin–streptomycin mixture were purchased from Lonza (Belgium). Foetal bovine serum (FBS), phorbol myristate acetate (PMA), prednisone, and the lipopolysaccharide (LPS) obtained from *Escherichia coli* 0111:B4 were purchased from Sigma-Aldrich (Germany). Instant ELISA TNF-α Kit (eBioscience, Vienna, Austria) was used to evaluate the production of TNF-α and IL-1β. The human monocytic leukemia cell line THP-1 was obtained from the European Collection of Cell Cultures (ECACC, Salisbury, UK).

The cells were cultivated at 37 °C in RPMI 1640 medium supplemented with 2 mM l-glutamine, 10% FBS, 100 U/mL of penicillin, and 100 μg/mL of streptomycin in a humidified atmosphere containing 5% of CO_2_. The growth medium was changed twice a week, when cells had reached a concentration of 5 × 10^5^–7 × 10^5^ in mL. The viability of cells was greater than 94% throughout the experiment. Stabilized cells were split into 24-well plates to get a concentration of 100,000 cells/mL, and the differentiation into macrophages was induced by phorbol myristate acetate (PMA). To promote differentiation of monocytes to macrophages, PMA was added to the final concentration 50 ng/mL and cells were stimulated for 24 h. In comparison with monocytes, differentiated macrophages tend to adhere on the bottom of cultivation plates. Next 24 h cells were incubated with fresh complete medium without PMA, then medium was aspirated, cells were washed with PBS and cultivated another 24 h in the serum-free RPMI 1640 medium. Prepared macrophages were consequently used for following experiments.

Differentiated macrophages were pretreated for 1 h with *Scorzonera* water methanolic extracts or compounds **1**–**16** dissolved in DMSO [25 μg/mL for extracts and 10 μM for compounds, respectively; this concentration lacks cytotoxic effect (data not shown)]. For comparison with conventional drugs, 1 μM prednisone dissolved in DMSO was used. Vehicle-treated cells contained the vehicle (DMSO) only. The concentration of DMSO was 0.1% in each well. The inflammatory response was triggered by adding LPS dissolved in water (1 μg/mL) to drug-pretreated macrophages, control cells remained without LPS stimulation, and the cells were incubated for another 24 h. After this time period, the medium was harvested and the concentration of TNF-α and IL-β was measured by using an Instant ELISA kits.

#### 3.5.3. Measurement of Inhibition of Activation of NF-κB

Differentiated macrophages were treated by tested extracts and LPS as was described above. Three hours after LPS adding, the cultivation medium was removed and cells were three times carefully washed by PBS (pH = 7.4) at room temperature (2 min per each washing). After this, cells were fixed (ice-cold acetone, 3 min) and washed two times by cold PBS. Incubation of cells in PBS containing 1% BSA (*w*/*v*) for 30 min and in the primary antibody (rabbit polyclonal Anti-NF-κB p65 antibody (product number ab7970), Abcam, UK) overnight at 4 °C followed. Used primary anti-p65 antibody recognizes C-terminal end of p65, which also serves as binding site for IκB-α. Hence, this antibody is able to bind to p65 only after IκB-α degradation. After incubation, cells were washed three times by PBS (5 min each wash) and incubated in secondary antibody (anti-rabbit IgG—FITC conjugate, PBS containing 1% BSA, *w*/*v*) for 1 h at room temperature. Finally, mixture was decanted, cells were washed by PBS (three times, 5 min, in dark), incubated with DAPI (0.5 μg/mL, PBS) for 1 min and washed by PBS. Cells were observed under a fluorescence microscope (Axioskop 40, Carl Zeiss, Germany) equipped by FITC and DAPI filters (Carl Zeiss). Photographs were taken using digital microscope camera (ProgRess MF, Jenoptik, Germany). NIS-element program (Czech Republic) was used for image processing—converting into color scale concentration images—and analysis—evaluation of intensity of emission. All chemicals used in this part of experiment were purchased from Sigma-Aldrich unless otherwise specified.

### 3.6. Statistical Analysis

All results were presented as the mean ± standard error of the mean (S.E.M.) or as a percentage. Analysis of variance (ANOVA) was used for the statistical analysis of the data. The Tukey HSD test (Tukey´s honestly significant difference test) was used to determine the significance. Results with *p* < 0.05 were considered to be statistically significant.

## 4. Conclusions

The anti-inflammatory potential of some *Scorzonera* extracts has been proved in *in vitro* TNF-α and IL-1β production inhibition assay on THP-1 macrophages, and supported by analysis of NF-κB nuclear translocation inhibition, but none of the compounds tested proved the activity in present study. More effort should be spend on the determination of active substances responsible for activity.

## Supplementary Materials

Supplementary materials can be accessed at: http://www.mdpi.com/1420-3049/21/1/43/s1.

## References

[1] Atanasov A.G., Waltenberger B., Pferschy-Wenzig E.M., Linder T., Wawrosch C., Uhrin P., Temml V., Wang L., Schwaiger S., Heiss E.H. (2015). Discovery and resupply of pharmacologically active plant-derived natural products: A review. Biotechnol. Adv..

[2] Yesilada E. (1997). Evaluation of the anti-inflammatory activity of the Turkish medicinal plant *Sambucus ebulus*. Chem. Nat. Prod..

[3] Bandgar B.P., Patil S.A., Totre J.V., Korbad B.L., Gacche R.N., Hote B.S., Jalde S.S., Chavan H.V. (2010). Synthesis and biological evaluation of nitrogen-containing benzophenone analogues as TNF-a and IL-6 inhibitors with antioxidant activity. Bioorg. Med. Chem. Lett..

[4] Leiro J.M., Varela M., Piazzon M.C., Arranz J.A., Noya M., Lamas J. (2010). The anti-inflammatory activity of the polyphenol resveratrol may be partially related to inhibition of tumour necrosis factor-α (TNF-α) pre-mRNA splicing. Mol. Immunol..

[5] Chauhan P.S., Satti N.K., Sharma V.K., Dutt P., Suri K.A., Bani S. (2011). Amelioration of inflammatory responses by chlorogenic acid via suppression of pro-inflammatory mediators. J. Appl. Pharm. Sci..

[6] Benbarek H., Deby-Dupont G., Deby C., Serteyn D. (2008). Direct stimulation of the oxidative activity of isolated equine neutrophils by TNF-α and IL-1β. Vet. Immunol. Immunopathol..

[7] Rao P.P.N., Kabir S.N., Mohamed T. (2010). Nonsteroidal Anti-Inflammatory Drugs (NSAIDs): Progress in Small Molecule Drug Development. Pharmaceuticals.

[8] Zelova H., Hosek J. (2013). TNF-α signalling and inflammation: Interactions between old acquaintances. Inflamm. Res..

[9] Cheng J., Chen M., Wallace D., Tith S., Arrhenius T., Kashiwagi H., Ono Y., Ishikawa A., Sato H., Kozono T. (2004). Discovery and structure-activity relationship of coumarin derivatives as TNF-α inhibitors. Bioorg. Med. Chem. Lett..

[10] Folmer F., Jaspars M., Solano G., Cristofanon S., Henry E., Tabudravu J., Black K., Green D.H., Küpper F.C., Aalbersberg W. (2009). The inhibition of TNF-α-induced NF-κB activation by marine natural products. Biochem. Pharmacol..

[11] Nam N.H., Jae Y.Y. (2009). NF-κB Inhibitory Activities of the Methanol Extracts and some Constituents therein of some Vietnamese Medicinal Plants. Sci. Pharm..

[12] Zidorn C., Ellmerer E.P., Sturm S., Stuppner H. (2003). Tyrolobibenzyls E and F from *Scorzonera humilis* and distribution of caffeic acid derivatives, lignans and tyrolobibenzyls in European taxa of the subtribe Scorzonerinae (Lactuceae, Asteraceae). Phytochemistry.

[13] Tsevegsuren N., Edrada R.A., Lin W., Ebel R., Torre C., Ortlepp S., Wray V., Proksch P. (2007). Biologically Active Natural Products from Mongolian Medicinal Plants *Scorzonera divaricata* and *Scorzonera pseudodivaricata*. J. Nat. Prod..

[14] Wang Y., Edrada-Ebel R.A., Tsevegsuren N., Sendker J., Braun M., Wray V., Lin W., Proksch P. (2009). Dihydrostilbene derivatives from the Mongolian medicinal plant *Scorzonera radiata*. J. Nat. Prod..

[15] Zidorn C., Ellmerer-Müller E.P., Stuppner H. (2000). Sesquiterpenoids from *Scorzonera hispanica* L.. Pharmazie.

[16] Auzi A.R., Hawisa N.T., Sherif F.M., Sarker S.D. (2007). Neuropharmacological properties of *Launaea resedifolia*. Rev. Bras. Farmacogn..

[17] Zhu Y., Wu Q., Hu P., Wu W. (2009). Biguaiascorzolides A and B: Two novel dimeric guaianolides with a rare skeleton, from *Scorzonera austriaca*. Food Chem..

[18] Sezik E., Yeşilada E., Tabata M., Honda G., Takaishi Y., Fujita T., Tanaka T., Takeda Y. (1997). Traditional medicine in Turkey VIII. Folk medicine in East Anatolia; Erzurum, Erzincan, Ağrı, Kars, Iğdır Provinces. Econ. Bot..

[19] Baytop T. (1999). Türkiye’de Bitkiler ile Tedavi (Theraphy with Medicinal Plants in Turkey).

[20] Donia A.M. (2013). Phytochemical and pharmacological studies on *Scorzonera alexandrina* Bioss. J. Saudi Chem. Soc..

[21] Bahadir Ö., Saltan Çitoğlu G., Šmejkal K., Dall'Acqua S., Özbek H., Cvačka J., Žemlička M. (2010). Analgesic Compounds from *Scorzonera latifolia* (Fisch. and Mey.) DC.. J. Ethnopharmacol..

[22] Küpeli Akkol E., Acıkara Bahadır Ö., Süntar İ., Çitoğlu Saltan G., Keleş H., Ergene B. (2011). Enhancement of wound healing by topical application of *Scorzonera* species: Determination of the constituents by HPLC with new validated reverse phase method. J. Ethnopharmacol..

[23] Küpeli Akkol E., Acıkara Bahadır Ö., Süntar İ., Ergene B., Çitoğlu Saltan G. (2012). Ethnopharmacological evaluation of some *Scorzonera* species: *In vivo* anti-inflammatory and antinociceptive effects. J. Ethnopharmacol..

[24] Süntar İ., Acıkara Bahadır Ö., Çitoğlu Saltan G., Keleş H., Ergene B., Küpeli Akkol E. (2011). *In vivo* and *In vitro* Evaluation of the Therapeutic Potential of Some *Scorzonera* Species as Wound healing Agent. Curr. Pharm. Des..

[25] Amdekar S., Roy P., Singh V., Kumar A., Singh R., Sharma P. (2012). Anti-Inflammatory Activity of Lactobacillus on Carrageenan-Induced Paw Edema in Male Wistar Rats. Int. J. Inflamm..

[26] Kim K.R., Jeong C.K., Park K.K., Choi J.H., Park J.H.Y., Lim S.S., Chung W.Y. (2010). Anti-Inflammatory Effects of Licorice and Roasted Licorice Extracts on TPA-Induced Acute Inflammation and Collagen-Induced Arthritis in Mice. J. Biomed. Biotechnol..

[27] Yeşilada E., Üstün O., Sezik E., Takaishi Y., Ono Y., Honda G. (1997). Inhibitory effects of Turkish folk remedies on inflammatory cytokines: Interleukin-1α, interleukin-1β and tumor necrosis factor α. J. Ethnopharmacol..

[28] Schwaiger S., Zeller I., Pölzelbauer P., Frotschnig S., Laufer G., Messner B., Pieri V., Stuppner H., Bernhard D. (2011). Identification and pharmacological characterization of the anti-inflammatory principal of the leaves of dwarf elder (*Sambucus ebulus* L.). J. Ethnopharmacol..

[29] Kaileh M., Berghe W.V., Boone E., Essawi T., Haegeman G. (2007). Screening of indigenous Palestinian medicinal plants for potential anti-inflammatory and cytotoxic activity. J. Ethnopharmacol..

[30] Siriwatanametanon N., Fiebich B.L., Efferth T., Prieto J.M., Heinrich M. (2010). Traditionally used Thai medicinal plants: *In vitro* anti-inflammatory, anticancer and antioxidant activities. J. Ethnopharmacol..

[31] Borchers A.T., Keen C.L., Stern J.S., Gershwin M.E. (2000). Inflammation and Native American medicine: The role of botanicals. Am J Clin Nutr.

[32] Dos Santos M.D., Almeida M.C., Lopes N.P., Souza G.E.P. (2006). Evaluation of the anti-inflammatory, analgesic and antipyretic activities of natural polyphenol chlorogenic acid. Biol. Pharm. Bull..

[33] Yonathan M., Asres K., Assefa A., Bucar F. (2006). *In vivo* anti-inflammatory and antinociceptive activities of *Cheilanthes farinose*. J. Ethnopharmacol..

[34] Hošek J., Šmejkal K., Parnham M. Flavonoids as Anti-inflammatory Agents. Encyclopedia of Inflammatory Diseases.

[35] Comalada M., Ballester I., Bailon E., Sierra S., Xaus J., Galvez J., Sanchez de Medina F., Zarzuelo A. (2006). Inhibition of pro-inflammatory markers in primary bone marrow-derived mouse macrophages by naturally occurring flavonoids: Analysis of the structure-activity relationship. Biochem. Pharmacol..

[36] Paul A.T., Gohil V.M., Bhutani K.K. (2006). Modulating TNF-α signaling with natural products. Drug Discov. Today.

[37] Yamamoto Y., Gaynor R.B. (2001). Therapeutic potential of inhibition of the NF-κB pathway in the treatment of inflammation and cancer. J. Clin. Investig..

[38] Jeong G.-S., Bae J.-S. (2014). Anti-Inflammatory Effects of Triterpenoids; Naturally Occurring and Synthetic Agents. Mini-Rev. Org. Chem..

[39] Sultana N., Saify Z.S. (2012). Naturally occurring and synthetic agents as potential anti-inflammatory and immunomodulants. Anti-Inflamm. Anti-Allergy Agents Med. Chem..

[40] Shen W., Qi R., Zhang J., Wang Z., Wang H., Hu C., Zhao Y., Bie M., Wang Y., Fu Y., Chen M., Lu D. (2012). Chlorogenic acid inhibits LPS-induced microglial activation and improves survival of dopaminergic neurons. Brain Res. Bull..

[41] Ku S.K., Zhou W., Lee W., Han M.S., Na M., Bae J.S. (2015). Anti-inflammatory effects of hyperoside in human endothelial cells and in mice. Inflammation.

[42] Srivastava P., Mohanti S., Bawankule D.U., Khan F., Shanker K. (2014). Effect of *Pluchea lanceolata* bioactives in LPS-induced neuroinflammation in C6 rat glial cells. Naunyn-Schmiedeberg’s Arch. Pharmacol..

[43] Chen J.-J., Tsai Y.-C., Hwang T.-L., Wang T.-C. (2011). Thymol, Benzofuranoid, and Phenylpropanoid Derivatives: Anti-inflammatory Constituents from *Eupatorium cannabinum*. J. Nat. Prod..

[44] Hashimoto T., Tori M., Asakawa Y. (1987). Three dihydroisocoumarine glucosides from *Hydrangea macrophylla* subsp. Serrate. Phytochemistry.

[45] Frank J.H., Powder-George Y.M., Ramsewak R.S., Reynolds W.F. (2012). Variable-Temperature ^1^H-NMR Studies on Two *C*-Glycosylflavones. Molecules.

[46] Pukalskas A., Venskutonis P.R., Dijkgraaf I., van Beek T.A. (2010). Isolation, identification and activity of natural antioxidants from costmary (*Chrysanthemum balsamita*) cultivated in Lithuania. Food Chem..

[47] Acikara Bahadir Ö., Saltan Citoglu G., Dall'Acqua S., Özbek H., Cvačka J., Žemlička M., Šmejkal K. (2014). Bioassay-guided isolation of the antinociceptive compounds motiol and β-sitosterol from *Scorzonera latifolia* root extract. Pharmazie.

[48] Paraschos S., Magiatis P., Kalpoutzakis E., Harvala C., Skaltsounis A.-L. (2001). Three New Dihydroisocoumarins from the Greek Endemic Species *Scorzonera cretica*. J. Nat. Prod..

[49] Acikara Bahadir Ö., Saltan Citoglu G., Dall’Acqua S., Šmejkal K., Cvačka J., Žemlička M. (2012). A new triterpene from *Scorzonera latifolia* (Fisch. and Mey.) DC.. Nat. Prod. Res..

