# Supplementary material for: Turkish Scorzonera Species Extracts Attenuate Cytokine Secretion via Inhibition of NF-κB Activation, Showing Anti-Inflammatory Effect in Vitro
Source: Molecules. 2015 Dec 30;21(1):43. doi: 10.3390/molecules21010043 (PMC6274538; doi:10.3390/molecules21010043)
Supplement: Supplementary file 1 [file molecules-21-00043-s001.pdf]

Supplementary Materials: Turkish *Scorzonera* Species Extracts Attenuate Cytokine Secretion via Inhibition of NF-κB Activation, Showing Anti-Inflammatory Effect *in Vitro*

Özlem Bahadır Acikara, Jan Hošek, Petr Babula, Josef Cvačka, Miloš Budešínský, Martin Dračinský, Gülçin Saltan İşcan, Daniela Kadlecová, Ludmila Ballová and Karel Šmejkal

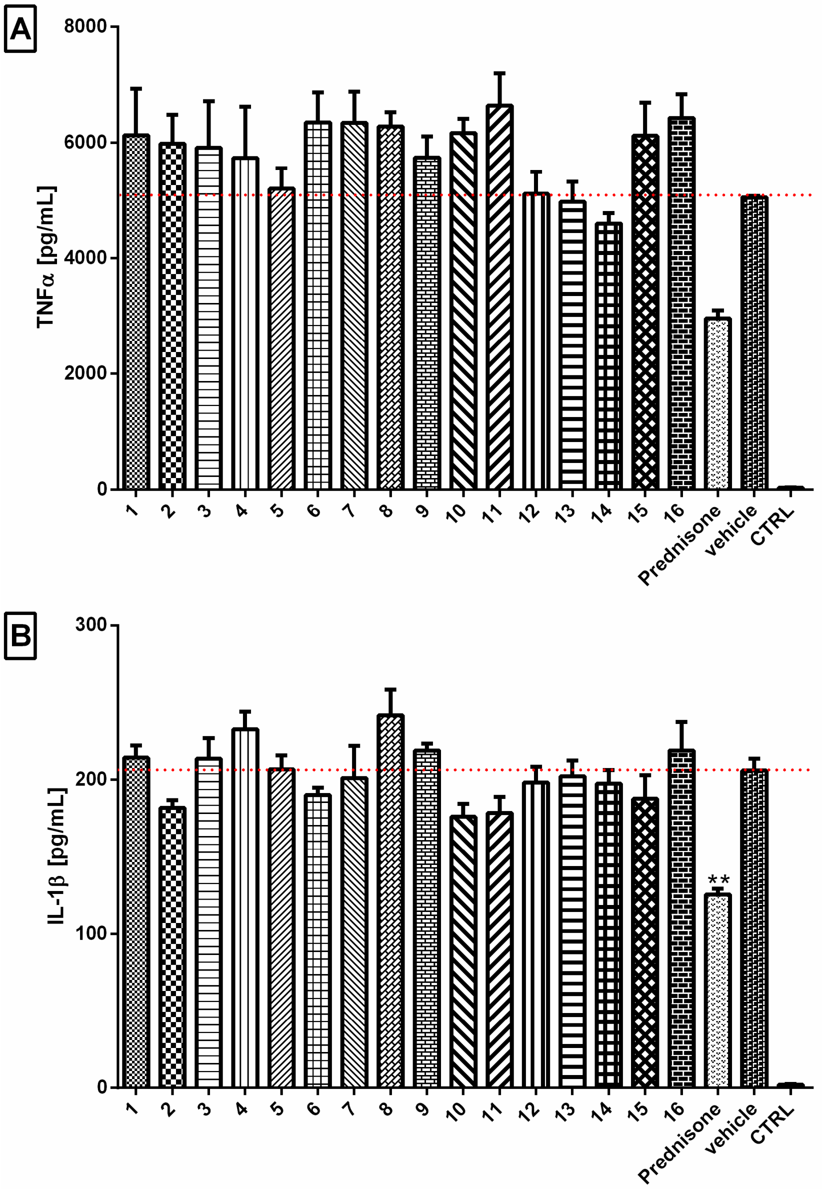

Figure S1. Cytokines production inhibitory activities of isolated compounds. The cells were pretreated with compounds **1–16** (10 μM), and prednisone (1 μM), or the vehicle (DMSO) only. After 1 h of the incubation, the inflammatory response was induced by LPS (except for the control cells). The secretion of TNF-*α* (**A**) and IL-1β (**B**) was measured 24 h after the LPS addition. The results are expressed as mean ± SE for three independent experiments. ** Significant difference in comparison to vehicle-treated cells ($p < 0.01$). Red dotted line indicates the cytokine level of DMSO treated cells.

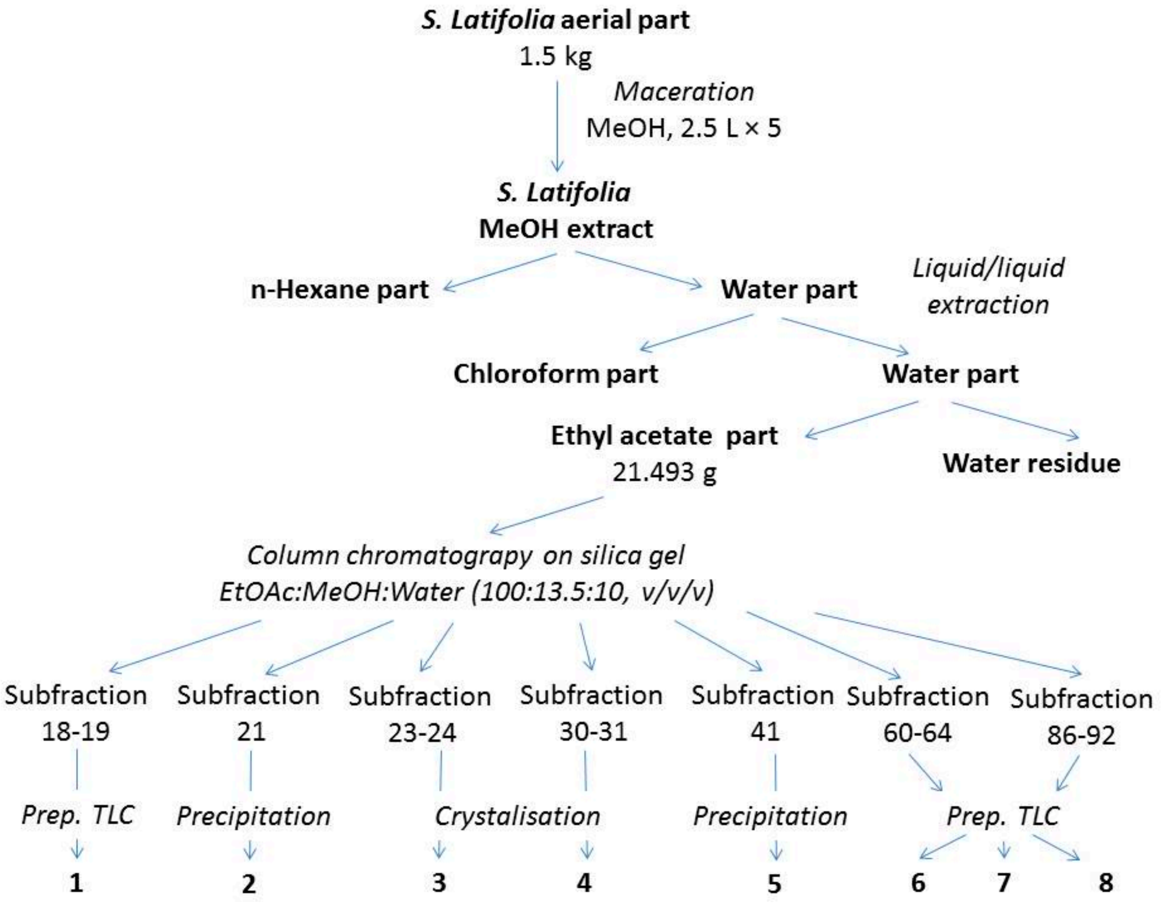

Figure S2. Scheme of separation of compounds **1**–**8**.
